# Using Telemedicine during the COVID-19 Pandemic: How Service Quality Affects Patients’ Consultation

**DOI:** 10.3390/ijerph191912384

**Published:** 2022-09-28

**Authors:** Xiaochen Liu, Zhen Xu, Xintao Yu, Tetsuaki Oda

**Affiliations:** 1 Graduate School of Technology Management, Ritsumeikan University, Ibaraki 567-8570, Japan; 2 School of Communication, East China University of Political Science and Law, Shanghai 201620, China; 3 School of Economics and Management, Liaoning University of Technology, Jinzhou 121001, China

**Keywords:** telemedicine platforms, signaling theory, patient consultation, service quality, health communication

## Abstract

The COVID-19 epidemic put pressure on the traditional healthcare system and offline consultation methods. Telemedicine platforms provide a more convenient and safer channel for online health communication. Based on the signaling theory, our study explores the impacts of three dimensions of physicians’ service quality (need fulfillment, security, and responsiveness) on online patient consultation on telemedicine platforms. A negative binomial model was used to test cross-sectional data of 2982 physicians obtained from Haodf.com. The results show the following: (1) the need fulfillment dimension variables positively affect online patient consultation; (2) the security dimension variables positively affect online patient consultation; (3) the responsiveness dimension variables positively affect online patient consultation. Our results contribute to the theoretical aspect of signaling theory and service quality in the context of telemedicine platforms and have several practical implications for telemedicine platform physicians and platform operators.

## 1. Introduction

With the rapid development of information technology (IT) and the rising need for medical services, the internet-based healthcare system is becoming increasingly common [1]. As one of the secure and simple platforms for physician–patient interactions, telemedicine platforms have been crucial in providing online patient consultation, especially during the outbreak of COVID-19 [2]. On the one hand, online patient consultation on telemedicine platforms decreased face-to-face contact between physicians and patients, contributing to viral (i.e., COVID-19) transmission and infection reduction [3]. On the other hand, telemedicine platforms can overcome geographic boundaries, expand the majority of individuals serviced, and offer patients online medical services, which facilitate patients contacting physicians and consulting with physicians about health problems and disease treatments at any time and from any location [4]. Although the contribution of online patient consultation on telemedicine platforms is outstanding in the current situation, the significant information asymmetry between patients and physicians might impair the quality of a patient’s healthcare [5]. Consequently, determining how patients solve information asymmetry to choose high-service-quality physicians has quickly become an important issue for telemedicine platforms.

Signaling theory is generally used to understand the attitudes of individuals and weaken information asymmetry issues [6]. Based on the signaling theory, signals are visible, extrinsic indications that may transmit trustworthy information about unobservable qualities, such as the service quality of sellers [7]. Signals are powerful means for sellers to convey service quality information, effectively reducing the information asymmetry between the buyers and sellers. Service quality is defined as a user’s assessment of a service’s overall brilliance or supremacy [8]. In the highly competitive service business, organizations have depended on service quality as a competitive advantage for decades [9]. In the context of telemedicine platforms, measuring the service quality provided by a physician is a significant challenge for patients. Due to the intangibility of services, it is more difficult to regulate their quality than it is with produced things [9]. As a result, academics began to focus on service quality in telemedicine platforms [10]. Cao et al. (2017) used service quality as a central route on the Elaboration Likelihood Model for examining patient consultation intention [11]. Wu (2018) identified that service quality affects customer perception by using a combination of web mining and structural equation modeling to analyze data from a telemedicine platform in China [12]. Thus, physicians’ service quality plays an extremely important role in choosing patient consultation.

Previous studies have generally considered service quality as a multidimensional concept. For instance, Gummerus’s study suggested that online service quality may be classified into four dimensions: need fulfillment, security, responsiveness, and user interface [13]. Akter et al. (2013) established a scale of online service quality with three dimensions: system quality, data quality, and interaction quality in health care [14]. However, previous telemedicine platform studies mostly regarded patients’ service quality as a one-dimensional variable, and few studies explored the multiple dimensions of patients’ service quality. For example, Shim and Jo (2020) examined how service quality affects users’ satisfaction and expectations of health information sites as part of information system quality [15]. Kaium et al. (2020) found that service quality significantly affects the continuance usage intention of mHealth among the rural elderly [16]. To fill the past gap, our research focuses on the impacts of multiple dimensions of physicians’ service quality on patient consultation.

Based on the signaling theory, our study explores the impacts of three dimensions of physicians’ service quality (need fulfillment, security, and service responsiveness) on online patient consultation on telemedicine platforms. We retrieved information from the Haodf.com website automatically using a Python crawler that can collect voluminous amounts of data from a website with speed and efficiency. Haodf.com is one of the most popular Chinese online healthcare communities (OHCs), with a large user base (physicians and patients). Finally, cross-sectional data on 2982 physicians were gathered. This paper examined the following two questions: (1) How should the dimensions of physicians’ service quality on telemedicine platforms be divided? (2) What is the impact of three dimensions of physicians’ service quality on online patient consultation on telemedicine platforms? Our findings enrich the research of signaling theory on telemedicine platforms and multiple dimensions of physicians’ service quality and help physicians attract patient consultation by optimizing the display of online service quality signals.

## 2. Theoretical Framework

### 2.1. Telemedicine Platforms

Telemedicine platforms are an extension of the traditional physician–patient relationship on the Internet, where patients can consult physicians on health issues and disease treatments anytime and anywhere [10]. On telemedicine platforms, patient consultation is a type of consultation where the physician and patient communicate from separate locations [17]. Online patient consultation is an innovative approach to meeting escalating medical demand, enabling users to overcome boundaries of geography and time to give more possibilities for choosing physicians throughout the globe, and its usage is increasing [18]. On telemedicine platforms, the amount of consultations is an essential measure of the activity of doctors on the site [19]. The main two benefits are immunization and efficiency. In terms of immunization, online patient consultation can avoid face-to-face contact between physicians and patients and reduce the risk of spreading COVID-19 [20]. Since COVID-19 is infectious pneumonia transmitted by the SARS-CoV-2 virus [21], individuals in proximity may readily transmit the virus; consequently, social isolation is critical to limiting its spread [20]. On the other hand, online patient consultation is very efficient for patients. For example, the shortage and overload of medical facilities have been a major concern during COVID-19, and good physicians have limited time and energy to work. Telemedicine platforms provide a new way to solve the contradiction between physicians and patients. Furthermore, the lockdown strategy involves the need to prove a negative COVID-19 test in the preceding 48 h [22], which is an extra expense and a huge waste of time. However, telemedicine platforms can solve this problem by breaking through time and space [4].

In the OHC industry, the significant information asymmetry between patients and physicians [23] has caused barriers for patients in deciding which physician to consult [5]. An OHC is a classic marketplace with information asymmetry, where physicians are more knowledgeable than patients [24]. Due to this information asymmetry issue, patients may not obtain high-quality care, as it is difficult for them to choose an appropriate service provider—a physician. Therefore, some researchers have been focusing on physician features and attempting to summarize which features can attract patients. For example, Liu et al. (2019) found that online and offline reviews affect telephone consultations significantly [25]. Gong et al. (2021) discovered that the physicians’ character traits and reputation significantly influence patient selection based on the trust theory [26]. Ouyang et al. (2022) found that the physician’s self-disclosed information positively influences a patient’s decision on telemedicine platforms [27]. However, previous research did not look into the quality of physicians’ service. In the online shopping context, the service quality of sellers is vital for consumer decisions because consumers prefer to establish or develop contact with sellers depending on their perceptions of their service quality [28]. Thus, our research question is as follows: How does the service quality of physicians affect a patient’s consultation decision? To overcome the information asymmetry problem in OHCs, it is vital to determine whether the service quality of physicians is effective for estimating information asymmetry concerns and influencing patient consultation decisions and behaviors.

### 2.2. Signaling Theory

Signaling theory is used to characterize the actions of two parties (individuals or organizations) when they have access to disparate pieces of information [29], where it offers a theoretical framework for how someone utilizes signals to transmit limited information about the service quality to another party in order to facilitate the purchase [30]. In other words, signaling theory is a powerful means for sellers to convey service quality information since signals are visible indications that might provide purchasers with information about a seller’s genuine service quality. Meanwhile, for buyers, before making a decision, buyers sometimes lack the knowledge necessary to appropriately judge the service quality of unknown suppliers; however, signals are visible, extrinsic indications that may transmit trustworthy information about the unobservable qualities, such as service quality of sellers [7]. As the result, when the information receiver understands the signal received, the consequent degree of service quality perceived will change appropriately. In summary, signaling theory is critical for understanding service quality.

Furthermore, on telemedicine platforms, since the two parties possess varying quantities and types of information, there is a large information imbalance between signalers and receivers [29]. For example, when the signaler (physician) has more knowledge than the receiver (patient), a possible conflict may arise [31]. Although the information asymmetry in telemedicine is more severe than that in in-person consultation [32], signaling theory postulates that signals may assist in reducing information asymmetry between physicians and patients during the pre-purchase stage of a transaction [33]. In this situation, signalers send signals to receivers to deliver a high-quality medical service [31]. A signaler (physician) transmits signals to the receiver (patient) in order to reflect the signaler’s service quality [6], and the receiver assesses the signaler’s quality and responds accordingly. Therefore, the signal sent by physicians influences the degree of information asymmetry and, thus, the actions of patients. When patients (receivers) seek consultation on telemedicine platforms, they intend to consult physicians, seeking various signals about their health condition from physicians (signalers) [34].

Some researchers studied the influence of physicians on patient decisions and behaviors from a signaling perspective in the OHC context. For instance, Shah et al. (2021) found that the market signals (online reputation) and seller signals (offline reputation and online effort) positively affect patients’ e-consultation choices [31]. Lu et al. (2021) have found that online signals (physicians’ log-in behaviors and online reviews) positively influence online patient consultation [35]. Gong et al. (2021) discovered that the physicians’ personal quality and the physicians’ online reputation positively affect the patients’ physician selection willingness in OHCs [26]. Ouyang et al. (2022) found that the physician’s self-disclosed information positively related to the patient’s decision in an OHC [27]. Previous studies generally investigated service quality from one or two dimensions. For example, Ren et al. (2021) found that the service quality of physicians positively affects internet-based economic returns based on social exchange theory [36]. Chen et al. (2022) found that both service perception and disconfirmation had a positive impact on patients’ positive emotional intensity in textual reviews based on expectation disconfirmation theory [10]. Still, there is a lack of research on how physicians’ service quality affects patient consultation decisions from multiple dimensions based on signaling theory. In light of previous research [37,38], this study employed signaling theory to explain the influence of physician service quality on online patient consultation selections.

## 3. Hypotheses

**Figure 1:**
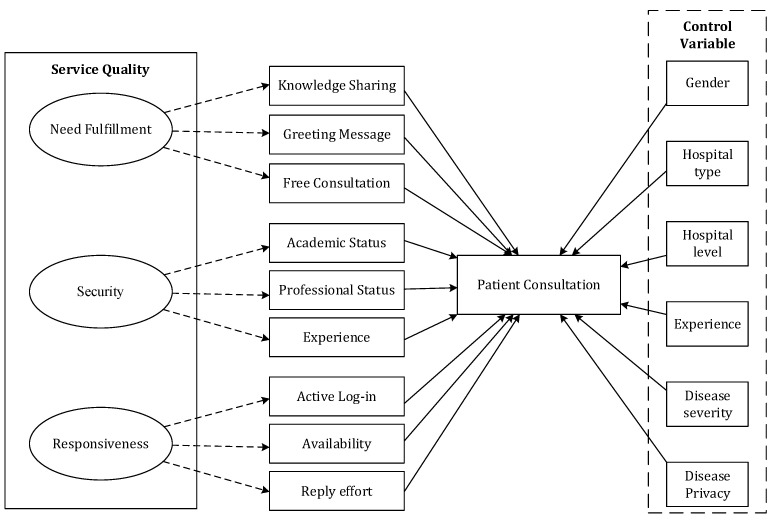
Research model.

In the context of telemedicine platforms, consumers visit physicians’ profiles to obtain information about the physician to determine whether the physician is capable of high service quality. Service quality is defined as the degree to which a service meets consumer expectations [39]. In the context of telemedicine platforms, the service quality of physicians, including their diagnosis and treatment skills, has a direct bearing on the chance of correct disease diagnosis and treatment and the health and safety of their patients [40]. Ren and Ma (2021) used the number of doctors’ answers from the patient consultation area to measure service quality [36]. However, previous telemedicine platform studies usually explored physicians’ service quality as a one-dimensional variable. Physicians display the relevant online signals concerning service quality to concerned patients through telemedicine platforms, influencing online patient consultation selections. Consequently, access to online physicians’ high-quality signals seems critical for patients, given their difficulty in obtaining relevant physicians’ service quality information due to the information asymmetry [41]. Gummerus’s study suggested that online service quality may be classified into four dimensions: need fulfillment, security, responsiveness, and user interface [13]. However, the user interface is a platform issue, not a physical issue, and we excluded the dimension of the user interface from our research. Thus, to explore the multiple dimensions of physicians’ service quality on telemedicine platforms, we adopt Gummerus’s study of online service quality with three dimensions (need fulfillment, security, and service responsiveness) [13]. As shown in Figure 1, we construct a research model of physicians’ online service quality and online patient consultation. It is based on signaling theory and explains how physicians’ service quality (need fulfillment, security, and responsiveness) influences online patient consultation.

### 3.1. Need Fulfillment and Online Patient Consultation

Need fulfillment refers to meeting client needs, a reliable indicator of both trust and contentment [13]. Previous studies on online communities in general and telemedicine platforms in particular reveal that patients require informational and emotional support [42]. Some health professionals (physicians) in telemedicine platforms may provide patients with information support, including free medical knowledge sharing and treatment advice out of sympathy, which may positively influence patient consultation [43]. For example, on telemedicine platforms, Zhang et al. (2022) suggested that online patient consultation was positively associated with knowledge sharing from physicians and a reflection of physician–patient relations [19]. Online patient consultation is a collaborative effort involving both parties. Due to the unpredictability of the disease, patients may require disease-related information anywhere and at any time, and internet health platforms are available, convenient, and anonymous [44]. On telemedicine platforms, some physicians share health knowledge through their free articles, which patients can use to discover the information they need. In addition, given the Internet’s easy accessibility and availability of information, many patients desire to be fully advised and involved in medical decision-making [45]. On telemedicine platforms, some physicians offer free consultations to answer patients’ simple questions, which may stimulate the next paid consultation.

Moreover, studies on online communities in general and telemedicine platforms in particular reveal that information gathering is only one of the numerous reasons people join such communities [46]. In addition to information, many users (patients) require emotional support [42], such as understanding, encouragement, empathy, affection, affirmation, validation, care, and concern [47]. In the context of telemedicine platforms, physicians express emotional support via their homepage greeting messages. Patients can determine whether the physician can provide emotional support by reviewing their greeting messages. Some physicians, for example, write encouraging and heartwarming remarks, while others write a few words about themselves or may not bother to write greeting messages for visitors. Since patients visit telemedicine platforms in search of emotional support, it has been shown that the presence of pleasant emotion in physicians’ information or messages influences patients’ decisions positively [2,27]. Therefore, we hypothesize the following:

**Hypothesis** **1.** *Knowledge sharing positively impacts online patient consultation*.

**Hypothesis** **2.** *Free consultation positively impacts online patient consultation*.

**Hypothesis** **3.** *Greeting message positively impacts online patient consultation*.

### 3.2. Security and Online Patient Consultation

Due to the features of healthcare, it is both vital and challenging for patients to find effective information to pick a reliable physician [48]. The features may be summarized as follows: Firstly, each patient’s illness is distinct [49]. Secondly, life and death are of the utmost significance [50]. Thirdly, there is considerable information asymmetry between physicians and patients, complicating matters [23]. Thus, on telemedicine platforms, selecting a reliable and experienced physician is usually the primary consideration for many patients [11,25,51]. Compared to traditional offline physician–patient interaction, telemedicine platforms allow patients to peruse the wealth of information available about different providers, and then patients select which physician to consult [52]. Thus, physicians, as signalers, give information to receivers (patients) (e.g., titles, workplaces, web-based activities, or reviews) [38], which can assist patients in selecting reliable physicians.

Security means freedom from danger, risk, and doubt [53], reflecting a good reputation and rich medical experience. Security might be a significant factor influencing the patient’s choice of online consultation, and it plays a crucial role in building trust [13]. Patients are more likely to pick the physician they trust [54]. Physicians with qualified and responsible information may be believed to provide high-level service quality, influencing patients’ decisions. A previous study shows that a patient’s decision is strongly influenced by a physician’s reputation (e.g., academic title and professional title) [51]. In general, a seller’s online reputation can positively affect their sales [55]. Similarly, on telemedicine platforms, patients prefer to choose physicians with a stellar online and offline reputation [31], which is mirrored by their professional and academic titles and wealth of experience. In general, physicians’ online and offline reputations serve as indicators of their overall capacity. Physicians’ medical abilities and experience show their capacity to demonstrate competence and discernment in various scenarios (Chung, 2012) and their extensive clinical experiences in making accurate diagnoses of their patients’ diseases [56]. Thus, patients expect their physicians to be experienced and highly professional [57]. In the telemedicine platform context, physicians’ academic and professional titles may reflect physicians’ professional expertise, and their experience reflects their ability to deliver personal care. Therefore, we propose the following:

**Hypothesis** **4.** *Academic title positively impacts online patient consultation*.

**Hypothesis** **5.** *Professional title positively impacts online patient consultation*.

**Hypothesis** **6.** *Experience positively impacts online patient consultation*.

### 3.3. Responsiveness and Online Patient Consultation

Responsiveness denotes the capacity of a service provider to reply swiftly to consumer requests and proposals and give support in the event of a problem [58]. In the context of online health platforms, physicians’ responsiveness is an essential metric that indicates consumers’ (patients’) impressions of the service provider’s capability and readiness to react to customer (patient) requests [13]. Consumers have recognized a prompt response as a characteristic of high-level service quality [59].

On telemedicine platforms, some of the physician’s webpage signals may be considered high responsiveness. Firstly, the physician’s active log-in behavior, availability, and reply effort may indicate his or her responsiveness. As one of a physician’s web-based behaviors, log-in behavior reflects the physician’s active involvement in the telemedicine platforms and their efforts. There is a relationship between activeness and the number of online patient consultations [60]. Patients are more likely to trust an active physician online [37,60]. Secondly, in offline healthcare contexts, some patients are inclined to have physicians available when necessary [61]. On telemedicine platforms, patients can verify physicians’ availability by physicians’ times for appointments. Physicians with a long time for appointments are considered more responsive when patients need them. Lastly, Deng et al. (2019) reveal that physicians’ online efforts positively contribute to the number of online patient consultations [51]. Service providers’ efforts are crucial to customers’ perceptions of service, and physicians’ behaviors will increase customers’ buy intentions or continuous purchase intents [62]. Effort refers to the quantity of energy "invested" in an activity per given period [63]. For physicians on telemedicine platforms, reply effort can be measured as the reply length of each consultation. When selecting a physician to consult with them about their health concerns, people favor those that exert the most effort online [51]. Therefore, we propose the following:

**Hypothesis** **7.** *Active log-in positively impacts online patient consultation*.

**Hypothesis** **8.** *Availability positively impacts online patient consultation*.

**Hypothesis** **9.** *Reply effort positively impacts online patient consultation*.

## 4. Methodology

### 4.1. Data and Measures

The dataset was obtained from Good Doctor Online (www.haodf.com), one of China’s largest online telemedicine platforms, established in 2006. Users can easily access the Good Doctor Online App, the PC website, the mobile website, and other platforms to handle a variety of medical difficulties, including online consultation services and offline treatment appointments. By October 2021, Good Doctor Online had registered over 240,000 physicians, 73% of these physicians working in large high-grade hospitals in China, and more than 740,000 patients had been served by the broad network of highly qualified medical providers [64]. The cross-sectional data on 2982 physicians were gathered between 6 and 8 April 2022 to better explore the patient online consultation behavior during the COVID-19 pandemic.

In this study, a Python crawler was used to collect nationwide physician information for research analysis, and no private information was involved. Since we could not obtain all of the data, we collected data on 14 diseases according to the severity of the disease [10]. High mortality diseases were as follows: (1) diabetes, (2) coronary heart disease, (3) hypertension, (4) Parkinson’s disease, (5) lung cancer, (6) liver cancer, and (7) breast cancer. Low mortality diseases were as follows: (1) infertility, (2) menstrual disorders, (3) prostatitis, (4) hepatitis B, (5) depression, (6) pharyngitis, and (7) pneumonia in children. Every physician on Good Doctor Online has a similar profile page that contains physician data, online consultation, appointment time, diagnostic evaluation, popular science area, personal achievements, etc. After physicians with missing values were eliminated, the final data included 2982 physicians. Detailed types and explanations of the data are shown in Table 1.

Table 1 shows the descriptive statistics, concluding with 16 variables, namely 1 dependent variable, 9 independent variables, and 6 control variables. The dependent variable is the total number of online patient consultations (Consult). On telemedicine platforms, the number of online patient consultations is an essential measure of physicians’ performance on the site [19]. The independent variables are the total number of articles shared by the physician (Sharing), length of the physician’s greeting messages (Greeting), total amount of free consultations (Free), academic title of physician (Aca_S), professional title of physician (Pro_S), years of online medical experience (Exp), last online log-in date (Log-in), time of appointment consultation that a physician has available (Ava*l*), and length of consultation reply from physicians (Reply). We also include control variables of physician-level and hospital-level characteristics that may influence the choice of patient consultation, including physicians’ gender (Gender), the hospital type with private or public (H_type), the hospital level with a scale from 1 to 3 (H_level), whether the hospital is a specialist hospital (H_Special), the mortality level of the disease (D_Severity), and the privacy level of the disease (D_Privacy).

### 4.2. Model Specification

This study considered both Poisson and negative binomial regression because the dependent variable is count data (total number of online patient consultations). Poisson regression is ruled out since the conditional variance exceeds the conditional expectation, and the data are excessively dispersed. To validate the model, this study examined the following main equation:

$$\begin{array}{ll} {\mathrm{Consult}}_{i}=\alpha_{0} & +\beta_{1}{\mathrm{logSharing}}_{i}+\beta_{2}{\mathrm{logGreeting}}_{i}+\beta_{3}{\mathrm{Free}}_{i}+\beta_{4}{\mathrm{Login}}_{i}+{\;\beta }_{5}{\mathrm{Aval}}_{i}\\ & +\beta_{6}{\mathrm{Reply}}_{i}+\beta_{7}\mathrm{Aca}\_S_{i}+\beta_{8}\mathrm{Pro}\_S_{i}+\beta_{9}{\mathrm{Expertise}}_{i}+\beta_{10}D\_{\mathrm{severity}}_{i}\\ & +\beta_{11}D\_{\mathrm{Privacy}}_{i}+\beta_{12}{\mathrm{Gender}}_{i}+\beta_{13}H\_{\mathrm{type}}_{i}+\beta_{9}H\_{\mathrm{level}}_{i}\\ & +\beta_{9}H\_{\mathrm{special}}_{i}+\mu_{i}+\varepsilon_{i} \end{array}$$

where denotes the city effects, is the constant term, and represents the residual error term. We apply log transformations to the Sharing with skewed distribution (skewness = 15.072) and Greeting with skewed distribution (skewness = 8.382) datasets. The equation estimates if dimension variables of physicians’ online service quality positively affect online patient consultation. Then, robustness check models separately use an alternative OLS regression model and the total number of patients’ visits as an alternative dependent variable. We take a log transformation on the dependent variable Consult since there is skewed distribution (skewness = 3.212) in OLS regression models. The total number of patients’ visits can be used as an alternative variable to the total number of consultations because patients can choose consultation only after visiting.

## 5. Result

Table 2 shows the correlation coefficients among all variables. The results show that Sharing (r = 0.403, *p* < 0.001), Greeting (r = 0.246, *p* < 0.001), Free (r = 0.407, *p* < 0.001), Login (r = 0.095, *p* < 0.001), Aval (r = 0.237, *p* < 0.001), Reply (r = 0.097, *p* < 0.001), Aca_S (r = 0.186, *p* < 0.001), Pro_S (r = 0.182, *p* < 0.001), and Exp (r = 0.367, *p* < 0.001) are significantly positively correlated with the dependent variable Consult. We evaluated the variance inflation factor (VIF) for estimating multicollinearity. Results indicate that the average VIF value is 2.24 (VIF < 10), indicating that multicollinearity is not a significant concern [65].

As shown in Table 3, we employed negative binomial regressions in main models 1 to 5, while models 6 and 7 showed OLS regressions and dependent variable substitution for the robustness checks. The control variables were analyzed in model 1. The result suggested that all control variables significantly affect online patient consultation. In models 2 to 4, three online service quality dimensions were introduced respectively for the robustness of all hypotheses. The results show that each service quality dimension has a significant and positive impact on online patient consultation.

In model 5, all control variables and independent variables were introduced. Model 5 firstly showed that the three variables of the need fulfilment dimension, namely Sharing (β = 0.135, *p* < 0.001), Free (β = 0.152, *p* < 0.001) and Greeting (β = 0.029, *p* < 0.01), have positive and significant effects on online patient consultation. Therefore, hypothesis 1 is supported, indicating that the physician’s knowledge sharing positively impacts online patient consultation. Hypothesis 2 is supported, indicating that the physician’s free consultation positively impacts online patient consultation. Hypothesis 3 is supported, which indicates that greeting messages of the physician positively affect online patient consultation.

Model 5 secondly showed that the three variables of the security dimension, namely Aca_S (β = 0.029, *p* < 0.01), Pro_S (β = 0.086, *p* < 0.01) and Exp (β = 0.092, *p* < 0.001), have positive and significant effects on online patient consultation. Therefore, hypothesis 4 is supported, which indicates that the academic title of the physician positively impacts online patient consultation. Hypothesis 5 is supported, which indicates that the professional title of the physician positively impacts online patient consultation. Hypothesis 6 is supported, which indicates that the online medical experience of the physician positively impacts online patient consultation.

Model 5 lastly showed that the three variables of the responsiveness dimension, namely Login (β = 0.174, *p* < 0.001), Aval (β = 0.029, *p* < 0.001) and Reply (β = 0.010, *p* < 0.01), have positive and significant effects on online patient consultation. Therefore, hypothesis 7 is supported, which indicates that the active log-in behavior of the physician positively impacts online patient consultation. Hypothesis 8 is supported, which indicates that the availability of the physician positively impacts online patient consultation. Hypothesis 9 is supported, indicating that the physician’s reply effort positively affects online patient consultation.

Thus, the research model can be accepted, showing that the dimensions of need fulfillment, security, and responsiveness positively influence online patient consultation. All hypotheses were supported (see Table 4).

In models 6 and 7, we employed two alternative evaluation methods as robustness checks. In model 6, we use OLS regression as an alternative evaluation method. In model 7, we use the total number of patients’ visits as a substitution dependent variable of patient consultation, because patients can choose consultation only after visiting. Consequently, the robustness check models have consistent coefficients with the main models.

## 6. Discussion

In the telemedicine platform context, the service quality of physicians has played a significant role [13]. Both patients and researchers are concerned about how to choose a physician who provides excellent care. Based on the signaling theory, this research explored the three dimensions (need fulfillment, security, and responsiveness) of online service quality of physicians in online patient consultation. As shown in Table 3, all hypotheses are supported.

Firstly, the physician’s need fulfillment dimension positively impacts online patient consultation. This study employed knowledge sharing, greeting messages, and free consultation on Online Good Physician to measure the physician’s need fulfillment ability. The result showed that all factors are positively significant, which means that physicians on telemedicine platforms are actively popularizing disease-related topics and articles, friendly expression of medical benevolence to patients, and some free consultations; satisfying these patient needs will increase the probability that the patients will select the physician on telemedicine platforms to some extent. The results are consistent with those of Gummerus et al. (2004), who found that need fulfillment positively affects trust. Trust is considered crucial in determining the patients’ willingness to choose [54]. Thus, the need fulfillment dimension positively impacts patient online consultation choices.

Secondly, the physician’s security dimension positively impacts online patient consultation. This study collected data on academic and professional titles and experience to measure the physician’s security. The result showed that all factors are positively significant, suggesting that the academic and professional titles and treatment experience of telemedicine platform physicians will positively affect patients’ tendency to choose physicians. These factors will make patients think that the physician has a high degree of safety and increase the probability of patients choosing the physician on telemedicine platforms. Therefore, the higher the physician’s grade index, the higher the probability of the physician being selected by patients. Patients are more likely to pick a physician if he or she has a high status and long professional experience [54]. Since physicians with high standing (academic and professional) understand the significance of security, they will offer a high level of security assurance given the significant dangers of healthcare services. Thus, the security dimension positively impacts patient online consultation choices.

Finally, the physician’s responsiveness positively impacts online patient consultation. This study adopted active log-in, availability, and reply effort as research data for analyzing physician responsiveness. The result showed that all factors are positively significant, indicating how often physicians log on to telemedicine platforms, how many half-day consultations they provide, and how efficiently they respond to patient questions all positively influence the patient’s choice. The increase in responsiveness makes patients believe that physicians are highly motivated, that their diseases can be diagnosed and treated quickly, and that problems can be solved quickly. Therefore, the higher the scale indicators of physicians’ responsiveness, the more inclined patients are to consult with the physicians. The results are consistent with previous studies [37,60]. In addition, physicians’ efforts are also crucial to patients’ intention to consult, which is consistent with a previous study [62]. Thus, physicians’ responsiveness is a critical service quality signal affecting online patient consultation.

## 7. Contribution

### 7.1. Theoretical Contribution

Firstly, this study contributes to the literature on online patient consultation on telemedicine platforms from the standpoint of signaling theory. The consultation of patients attracted substantial interest from researchers. For example, using a web crawler, Ouyang et al. (2022) evaluated the impact of physicians’ self-disclosed information on patients’ decisions on telemedicine platforms [27]. Liu et al. (2019) used a web crawler to collect Good Doctor Online data to compare online and offline reviews of telephone consultations [25]. However, in the situation of telemedicine platforms, there is a large information imbalance between signalers and receivers [29]. Physicians’ online signals can influence the degree of information asymmetry and thus the actions of patients. Thus, the signaling theory is beneficial for understanding patients’ choices and reducing information asymmetry [6].

Secondly, this research contributes to the literature on service quality by exposing the signaling mechanism of physicians’ service quality in online patient consultation. Service quality plays a key part in developing competitiveness-enhancing strategic plans [9]. Health services are intangible, diverse, and interdependent, and each patient’s illness is unique [49]. In this circumstance, service quality is of utmost importance. However, little research explores the multiple dimensions of physicians’ online service quality to our knowledge. Most telemedicine platform studies recognize physicians’ online service quality as a signal-dimension variable [11]. In this research, three signals of physicians’ online service quality, need fulfillment, security, and responsiveness, were found to significantly influence online patient consultations on telemedicine platforms. Our research fills the gap, exploring the multiple dimensions of physicians’ online service quality and the effects of dimensions on online patient consultation.

### 7.2. Practical Contribution

Firstly, physicians should comprehend the consultation selection criteria of patients. Our findings indicate that the service quality of physicians (need fulfillment, security, and responsiveness) positively influences online patient consultation. When picking a physician to consult with, people place a premium on a physician’s ability to meet their needs, provide security, and be responsive. Therefore, physicians should be aware of the signals optimizing physicians’ online service quality, for instance, by evaluating physicians’ knowledge sharing, greeting messages, and free consultation to determine if they can meet patients’ needs; via evaluating physicians’ experience and academic and professional titles to ascertain their security level; or by evaluating physicians’ active log-in behavior, availability, and reply effort on their homepage to assess if they can respond to patients’ questions promptly. Consequently, physicians should examine these dimensions to release high online service quality signals to attract patients’ choices.

Secondly, platform operators may make high-quality information more accessible to patients and motivate physician groups. Our findings indicate that service quality is a factor that patients consider when selecting a physician. Thus, managers may implement strategies to direct more people to the physician profiles with extensive knowledge-sharing and free consultations and urge physicians to send a kind greeting message. For instance, patients might be referred to physicians with more articles. Meanwhile, managers might direct patients to see physicians with extensive expertise and prestigious academic and professional designations. In addition, managers should propose physicians who can respond rapidly and actively log in online, in addition to ensuring that patients receive timely care and that physicians who are engaged online receive many more patients.

## 8. Limitations and Future Research

Firstly, the results are constrained by the use of data from a single Chinese telemedicine platform, Haodf.com. In the future, concurrently gathering data from physicians on many platforms is required to validate the study model further. Moreover, considering applicability restrictions to other nations, future studies could also include data from several nations. Secondly, it is difficult to differentiate between patients who consult online and those who browse online but consult offline. In this study, we relied on the number of patients and visits acquired by the platform, which may have led to the loss of certain data. Future studies may discover a solution to include additional data. Thirdly, only the cross-sectional data of the current period were captured by crawler technology in this study. Future studies may collect long-term panel data for more effects, such as about half a year of physicians’ home pages. Finally, this study gathered data from a Chinese OHC platform during the COVID-19 pandemic. In the future, researchers may continue to investigate the use behaviors deeply after this pandemic.

## 9. Conclusions

Online patient consultation criteria have remained a source of frustration for patients and interest for scholars. This research used signaling theory to evaluate the impact of service quality in three dimensions on online patient consultation. Based on the cross-sectional data of 2982 physicians from a Chinese telemedicine platform, this research reveals that physicians’ service quality (need fulfillment, security, and responsiveness) positively influences online patient consultation. This study also provides theoretical contributions to signaling theory and service quality on telemedicine platforms and practical implications for physicians and platform operators.

## Figures and Tables

**Table 1:** Descriptive statistics.

Variables	Description	Mean	S.D.	Min	Max
Dependent Variable					
Consult	Total number of online patient consultations	4581.30	5671.15	73.00	71,678.00
Independent Variable					
Sharing	Number of shared health articles	53.76	200.93	0.00	5720.00
Greeting	The length of the doctor’s greeting message	113.94	202.95	0.00	3975.00
Free	Total number of free online consultations	2.93	2.02	0.00	10.65
Aca_S	Academic title of physician titles was classified into four levels; 1 = teaching assistant, 2 = lecturer, 3 = associate professor, 4 = professor	1.62	1.68	0.00	4.00
Pro_S	The medical titles of the physician were stratified into 4 stages; 1 = the resident physician, 2 = the attending physician, 3 = associate chief director, 4 = chief director.	3.25	0.74	1.00	4.00
Exp	The number of years that a physician had conducted online consultation on the platform	8.01	3.55	1.00	14.00
Login	Last online date; 1 = over 1 day ago, 2 = within a day, 3 = today	2.37	0.59	1.00	3.00
Aval	The number of half-day consultations that a physician has available	7.18	5.34	0.00	35.00
Reply	The average number of responses from doctors	4.65	3.53	0.00	65.75
Control Variable					
Gender	Dummy variable indicating physician gender; 0 = male, 1 = female	0.34	0.47	0.00	1.00
H_type	Dummy variable indicating the hospital type; 0 = private, 1 = public	0.99	0.08	0.00	1.00
H_level	Hospital level: the scale of 1 to 3, with 1 being the lowest (1A or 1B) and 3 the highest (3A or 3B hospitals)	2.99	0.12	1.00	3.00
H_Special	Dummy variable indicating whether the hospital is a specialized hospital; 0 = specialized, 1 = general	0.67	0.47	0.00	1.00
D_severity	Dummy variable indicating the mortality of the disease; 0 = low, 1 = high	0.37	0.48	0.00	1.00
D_Privacy	Dummy variable indicating the privacy level of the disease; 0 = low, 1 = high	0.22	0.42	0.00	1.00

**Table 2:** Correlation coefficient matrix.

	1	2	3	4	5	6	7	8	9	10	11	12	13	14	15	16
Consult	1.000															
logSharing	0.403 ***	1.000														
	(0.000)															
logGreeting	0.246 ***	0.435 ***	1.000													
	(0.000)	(0.000)														
Free	0.407 ***	0.344 ***	0.195 ***	1.000												
	(0.000)	(0.000)	(0.000)													
Login	0.095 ***	0.037 *	0.015 *	0.070 ***	1.000											
	(0.000)	(0.044)	(0.423)	(0.000)												
Aval	0.237 ***	0.121 ***	0.046 *	0.112 ***	−0.038 *	1.000										
	(0.000)	(0.000)	(0.011)	(0.000)	(0.040)											
Reply	0.097 ***	0.117 ***	0.053 **	0.085 ***	0.065 ***	0.018 *	1.000									
	(0.000)	(0.000)	(0.004)	(0.000)	(0.000)	(0.329)										
Aca_S	0.186 ***	0.159 ***	0.136 ***	0.101 ***	−0.002	0.028 *	−0.065 ***	1.000								
	(0.000)	(0.000)	(0.000)	(0.000)	(0.912)	(0.128)	(0.000)									
Pro_S	0.182 ***	0.140 ***	0.124 ***	0.075 ***	−0.014 *	0.067 ***	−0.111 ***	0.448 ***	1.000							
	(0.000)	(0.000)	(0.000)	(0.000)	(0.435)	(0.000)	(0.000)	(0.000)								
Expertise	0.367 ***	0.358 ***	0.274 ***	0.124 ***	−0.017 *	0.097 ***	−0.074 ***	0.352 ***	0.419 ***	1.000						
	(0.000)	(0.000)	(0.000)	(0.000)	(0.341)	(0.000)	(0.000)	(0.000)	(0.000)							
D_severity	−0.111 ***	−0.009	0.019 *	−0.064 ***	−0.005	−0.218 ***	−0.005	0.068 ***	0.081 ***	0.021 *	1.000					
	(0.000)	(0.632)	(0.290)	(0.000)	(0.771)	(0.000)	(0.793)	(0.000)	(0.000)	(0.252)						
D_privacy	0.075 ***	−0.015 *	−0.047 **	0.039 *	−0.078 ***	0.256 ***	−0.000	−0.059 **	−0.027 *	−0.020 *	−0.407 ***	1.000				
	(0.000)	(0.407)	(0.010)	(0.031)	(0.000)	(0.000)	(0.993)	(0.001)	(0.142)	(0.278)	(0.000)					
Gender	−0.085 ***	−0.184 ***	−0.124 ***	−0.093 ***	−0.030 *	0.180 ***	0.022 *	−0.074 ***	0.054 **	−0.135 ***	−0.160 ***	0.230 ***	1.000			
	(0.000)	(0.000)	(0.000)	(0.000)	(0.105)	(0.000)	(0.238)	(0.000)	(0.003)	(0.000)	(0.000)	(0.000)				
H_type	−0.014 *	−0.065 ***	−0.016 *	−0.024 *	0.005	−0.087 ***	−0.018 *	0.022 *	0.002	0.035 *	0.039 *	−0.090 ***	−0.033 *	1.000		
	(0.453)	(0.000)	(0.389)	(0.196)	(0.796)	(0.000)	(0.314)	(0.238)	(0.926)	(0.054)	(0.033)	(0.000)	(0.070)			
H_level	0.021 *	−0.028 *	−0.002	−0.022 *	0.003	−0.045 *	0.004	0.064 ***	0.001	0.067 ***	0.049 **	−0.077 ***	−0.061 ***	0.385 ***	1.000	
	(0.245)	(0.129)	(0.930)	(0.233)	(0.887)	(0.014)	(0.815)	(0.001)	(0.941)	(0.000)	(0.008)	(0.000)	(0.001)	(0.000)		
H_special	−0.032 *	0.038 *	0.018 *	0.016 *	−0.013 *	−0.059**	−0.022 *	0.175 ***	0.024 *	0.006	0.095 ***	−0.028 *	−0.063 ***	0.061 ***	0.029 *	1.000
	(0.080)	(0.036)	(0.331)	(0.380)	(0.493)	(0.001)	(0.235)	(0.000)	(0.184)	(0.760)	(0.000)	(0.125)	(0.001)	(0.001)	(0.115)	

*p*-values in parentheses. * *p* < 0.05, ** *p* < 0.01, *** *p* < 0.001.

**Table 3:** Regression result.

	Main Models					Robustness Models	
	Model 1	Model 2	Model 3	Model 4	Model 5	Model 6	Model 7
Constant	7.898 ***	5.535 ***	7.422 ***	6.988 ***	4.887 ***	4.722 ***	8.988 ***
	(0.51)	(0.31)	(0.53)	(0.52)	(0.29)	(0.31)	(0.40)
D_severity	−0.280 ***	−0.283 ***	−0.354 ***	−0.226 ***	−0.291 ***	−0.259 ***	−0.250 ***
	(0.05)	(0.04)	(0.04)	(0.05)	(0.04)	(0.03)	(0.05)
D_privacy	0.152**	0.075 *	0.132 *	0.077 *	0.025	0.085*	0.145 **
	(0.06)	(0.05)	(0.06)	(0.05)	(0.04)	(0.04)	(0.05)
Gender	−0.301 ***	−0.008	−0.184 ***	−0.332 ***	−0.022	−0.037 *	−0.027
	(0.05)	(0.04)	(0.04)	(0.04)	(0.04)	(0.03)	(0.04)
H_type	−0.232 *	0.222 *	−0.170	−0.077	0.213 *	0.246 *	0.511 *
	(0.34)	(0.21)	(0.34)	(0.36)	(0.18)	(0.20)	(0.20)
H_level	0.341 *	0.388 ***	0.025	0.218 *	0.154 *	0.041	−0.019
	(0.17)	(0.12)	(0.19)	(0.19)	(0.11)	(0.11)	(0.15)
H_special	−0.071 *	−0.092 *	−0.096 *	−0.042 *	−0.081 *	−0.082 *	−0.114 **
	(0.05)	(0.04)	(0.04)	(0.04)	(0.04)	(0.03)	(0.04)
logSharing		0.208 ***			0.135 ***	0.127 ***	0.238 ***
		(0.01)			(0.01)	(0.01)	(0.02)
logGreeting		0.059 ***			0.029 **	0.044 ***	0.038 ***
		(0.01)			(0.01)	(0.01)	(0.01)
Free		0.167 ***			0.152 ***	0.166 ***	0.154 ***
		(0.01)			(0.01)	(0.01)	(0.01)
Aca_S			0.044***		0.029 **	0.034**	0.043 **
			(0.01)		(0.01)	(0.01)	(0.01)
Pro_S			0.071*		0.086 **	0.108 ***	0.136 ***
			(0.03)		(0.03)	(0.03)	(0.03)
Exp			0.130***		0.092 ***	0.097 ***	0.255 ***
			(0.01)		(0.01)	(0.01)	(0.01)
Login				0.213 ***	0.174 ***	0.164 ***	0.148 ***
				(0.04)	(0.03)	(0.03)	(0.03)
Aval				0.050 ***	0.029 ***	0.026 ***	0.027 ***
				(0.00)	(0.00)	(0.00)	(0.00)
Reply				0.017 ***	0.010 **	0.015 ***	0.024 ***
				(0.00)	(0.00)	(0.00)	(0.01)
City dummies	Yes	Yes	Yes	Yes	Yes	Yes	Yes
Wald chi-square (p)	0	0	0	0	0		0
*N*	2982	2982	2982	2982	2982	2982	2982

Standard errors in parentheses. * *p* < 0.05, ** *p* < 0.01, *** *p* < 0.001.

**Table 4:** Test of hypotheses.

Hypotheses	Results
**H1.** *Knowledge sharing positively impacts online patient consultation.*	Supported
**H2.** *Free consultation positively impacts online patient consultation.*	Supported
**H3.** *Greeting message positively impacts online patient consultation.*	Supported
**H4.** *Academic title positively impacts online patient consultation.*	Supported
**H5.** *Professional title positively impacts online patient consultation.*	Supported
**H6.** *Experience positively impacts online patient consultation.*	Supported
**H7.** *Active log-in positively impacts online patient consultation.*	Supported
**H8.** *Availability positively impacts online patient consultation.*	Supported
**H9.** *Reply effort positively impacts online patient consultation.*	Supported

## Data Availability

Data are available on request due to privacy and ethical restrictions. The data presented in this study are available on request from the corresponding author.
